# LPI-deepGBDT: a multiple-layer deep framework based on gradient boosting decision trees for lncRNA–protein interaction identification

**DOI:** 10.1186/s12859-021-04399-8

**Published:** 2021-10-04

**Authors:** Liqian Zhou, Zhao Wang, Xiongfei Tian, Lihong Peng

**Affiliations:** 1 School of Computer Science, Hunan University of Technology, No. 88, Taishan West Road, Tianyuan District, Zhuzhou, China; 2 College of Life Sciences and Chemistry, Hunan University of Technology, No. 88, Taishan West Road, Tianyuan District, Zhuzhou, China

**Keywords:** lncRNA–protein interaction, Multiple-layer deep architecture, Gradient boosting decision tree

## Abstract

**Background:**

Long noncoding RNAs (lncRNAs) play important roles in various biological and pathological processes. Discovery of lncRNA–protein interactions (LPIs) contributes to understand the biological functions and mechanisms of lncRNAs. Although wet experiments find a few interactions between lncRNAs and proteins, experimental techniques are costly and time-consuming. Therefore, computational methods are increasingly exploited to uncover the possible associations. However, existing computational methods have several limitations. First, majority of them were measured based on one simple dataset, which may result in the prediction bias. Second, few of them are applied to identify relevant data for new lncRNAs (or proteins). Finally, they failed to utilize diverse biological information of lncRNAs and proteins.

**Results:**

Under the feed-forward deep architecture based on gradient boosting decision trees (LPI-deepGBDT), this work focuses on classify unobserved LPIs. First, three human LPI datasets and two plant LPI datasets are arranged. Second, the biological features of lncRNAs and proteins are extracted by Pyfeat and BioProt, respectively. Thirdly, the features are dimensionally reduced and concatenated as a vector to represent an lncRNA–protein pair. Finally, a deep architecture composed of forward mappings and inverse mappings is developed to predict underlying linkages between lncRNAs and proteins. LPI-deepGBDT is compared with five classical LPI prediction models (LPI-BLS, LPI-CatBoost, PLIPCOM, LPI-SKF, and LPI-HNM) under three cross validations on lncRNAs, proteins, lncRNA–protein pairs, respectively. It obtains the best average AUC and AUPR values under the majority of situations, significantly outperforming other five LPI identification methods. That is, AUCs computed by LPI-deepGBDT are 0.8321, 0.6815, and 0.9073, respectively and AUPRs are 0.8095, 0.6771, and 0.8849, respectively. The results demonstrate the powerful classification ability of LPI-deepGBDT. Case study analyses show that there may be interactions between GAS5 and Q15717, RAB30-AS1 and O00425, and LINC-01572 and P35637.

**Conclusions:**

Integrating ensemble learning and hierarchical distributed representations and building a multiple-layered deep architecture, this work improves LPI prediction performance as well as effectively probes interaction data for new lncRNAs/proteins.

## Introduction

Long noncoding RNAs (lncRNAs) are a class of important noncoding RNAs with the length more than 200 nucleotides. The class of RNAs have been reported to have dense associations with multiple biological processes including RNA splicing, transcriptional regulation, and cell cycle [1–3]. More importantly, the mutations and dysregulations of lncRNAs have important affects on multiple cancers [4, 5], for instance, lung cancer [6], colon cancer [7], and prostate cancer [8]. For example, lncRNAs UCA1, PCA3, and HOTAIR have been used as possible biomarkers of bladder cancer detection, prostate cancer aggressiveness, and hepatocellular carcinoma recurrence, respectively [9–11]. Although lncRNAs have been intensively investigated, functions and molecular mechanisms of lncRNAs still largely remain elusive [2, 12]. Recent researches have revealed that lncRNAs densely link to the corresponding binding-proteins. Therefore, the identification of the binding proteins for lncRNAs is urgent for better understanding the biological functions and molecular mechanisms of lncRNAs [1].

Although wet experiments for lncRNA–protein Interaction (LPI) discovery have been designed, computational methods are appealing to infer the relevances between lncRNAs and proteins [13]. The computational methods can be roughly divided into two categories: network-based methods and machine learning-based methods. Network-based LPI inference methods integrated various biological data and designed network propagation methods to find potential LPIs in the heterogeneous lncRNA–protein network. For example, Li et al. [14] proposed a random walk with restart-based LPI prediction model. Zhou et al. [15] took miRNAs as mediators to predict LPIs in a heterogeneous network (LPI-HNM). Yang et al. [16] used the HeteSim algorithm to compute the associated scores between lncRNAs and proteins. Zhao et al. [17], Ge et al. [18], and Xie et al. [19] explored a few bipartite network projection-based recommendation techniques to compute the interaction probabilities between lncRNAs and proteins. Zhang et al. [20] explored a novel LPI prediction framework combining a linear neighborhood propagation algorithm. Zhou et al. [21] combined similarity kernel fusion and Laplacian regularized least squares to find unobserved LPIs (LPI-SKF).

Machine learning-based LPI inference methods characterized the biological features of lncRNAs and proteins and exploited machine learning algorithms to probe LPI candidates [22]. Machine learning-based LPI prediction methods contain matrix factorization techniques and ensemble learning techniques [23]. Matrix factorization-based LPI prediction approaches used various matrix factorization techniques. Liu et al. [24] identified new LPIs combing neighborhood regularized logistic matrix factorization. Zhao et al. [25] inferred LPI candidates combining the neighborhood regularized logistic matrix factorization model and random walk. Zhang et al. [26] proposed a graph regularized nonnegative matrix factorization method to uncover unobserved LPIs.

Ensemble learning-based LPI inference methods utilized diverse ensemble techniques. Zhang et al. [27] exploited an ensemble learning model to discover the interactions between lncRNAs and proteins. Liu et al. [24] designed three ensemble strategies to predict LPIs based on support vector machine, random forest and extreme gradient boosting, respectively. Deng et al. [1] extracted HeteSim features and diffusion features of lncRNAs and proteins and constructed a gradient tree boosting-based LPI prediction algorithm (PLIPCOM). Fan and Zhang [28] explored a stacked ensemble-based LPI classification model via logistical regression (LPI-BLS). Deng et al. [29] proposed a gradient boosted regression tree for finding possible LPIs. Wekesa et al. [30] designed a categorical boosting-based LPI discovery framework (LPI-CatBoost). In addition, deep learning (such as deep graph neural network [31]) is increasingly developed to identify LPI candidates.

Computational methods effectively identified potential LPIs. However, there are a few problems to solve. First, the majority of computational models were evaluated on one dataset, which may result in predictive bias. Second, they were not used to infer potential proteins (or lncRNAs) associated with a new lncRNA (or protein). Finally, their prediction performance need to further improve.

To solve the above problems, in this study, inspired by Gradient Boosting Decision Trees (GBDT) provided by Feng et al. [32], we exploit a multiple-layer Deep structure with GBDT to predict unobserved LPIs (LPI-deepGBDT). First, five LPI datasets are constructed. Second, lncRNA and protein features are extracted by Pyfeat and BioProt, respectively. Third, a feature vector is built to represent an lncRNA–protein pair. Finally, a multiple-layer deep architecture integrating tree ensembles and hierarchical distributed representations is developed to classify lncRNA–protein pairs.

The remaining of this manuscript is organized as follows. “Materials and methods” section describes data resources and the LPI-deepGBDT framework. “Results” section illustrates the results from a series of experiments. “Discussion and further research” section discusses the LPI-deepGBDT method and provides directions for further research.

## Materials and methods

### Data preparation

In this manuscript, we collect three human LPI datasets and two plant LPI datasets. Dataset 1 provided by Li et al [14] contains 3,487 LPIs from 938 lncRNAs and 59 proteins. 3,479 LPIs between 935 lncRNAs and 59 proteins are finally obtained by removing the lncRNAs without sequence information in the NONCODE [33], NPInter [34] and UniProt [35] databases.

Dataset 2 build by Zheng et al. [36] contains human 4,467 LPIs between 1,050 lncRNAs and 84 proteins. 3,265 LPIs from 885 lncRNAs and 84 proteins are extracted after removing the lncRNAs without any sequence information. Dataset 3 constructed by Zhang et al. [20] contains 4,158 LPIs between 990 lncRNAs and 27 proteins.

Datasets 4 provides 948 Arabidopsis thaliana LPIs from 109 lncRNAs and 35 proteins. Dataset 5 provides 22,133 Zea mays LPIs from 1,704 lncRNAs and 42 proteins. The sequence information of two entities is downloaded from the PlncRNADB database [37] and LPIs are extracted at http://bis.zju.edu.cn/PlncRNADB/. The details are described in Table 1.

**Table 1:** The statistics of LPI information

Dataset	lncRNAs	Proteins	LPIs
Dataset 1	935	59	3479
Dataset 2	885	84	3265
Dataset 3	990	27	4158
Dataset 4	109	35	948
Dataset 5	1704	42	22,133

We denote an LPI network via a matrix *Y*:

1 $$\begin{aligned} {y_{ij}} = \left\{ {\begin{array}{*{20}{l}} {1,{\mathrm {\, if \,\,lncRNAs \,\,}}{l_i}{\mathrm {\,\, interacts \,\,with \,\,protein\,\, }}{p_j}}\\ {0,{\mathrm { \, otherwise}}} \end{array}} \right. \end{aligned}$$

### Overview of LPI-deepGBDT

**Fig. 1:**
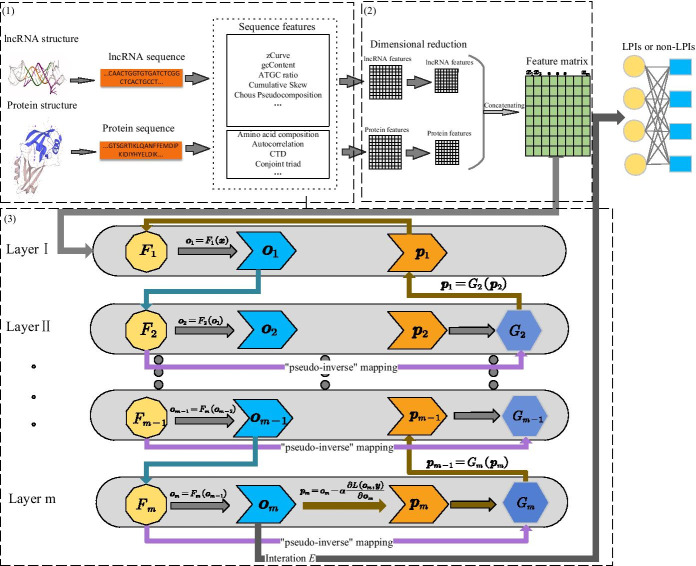
The flowchart of the LPI-deepGBDT framework. (1) Feature selection. (2) Dimension reduction. (3) Classification

In this study, we develop a feed-forward deep framework to infer new LPIs. Figure 1 describes the flowchart of LPI-deepGBDT. As shown in Fig. 1, the LPI-deepGBDT framework consists of three main processes after LPI datasets are built. (1) Feature extraction. Pyfeat [38] and BioProt [39] are used to extract the original features for lncRNAs and proteins. (2) Feature selection. The lncRNA and protein features are reduced into two *d*-dimensional vector based on dimensional reduction analysis with Principle Component Analysis (PCA). The two vectors are then concatenated to depict lncRNA–protein pairs. (3) Classification. A multiple-layer deep structure, composed of forward mapping and inverse mapping, is developed to classify lncRNA–protein pairs.

### Feature extraction

#### Feature extraction of lncRNAs

Pyfeat [38] is widely applied to generate numerical features via sequence information. In this study, we use Pyfeat to obtain lncRNA features and represent an lncRNA as a 3, 051-dimensional vector. The details are shown in Table 2.

**Table 2:** The lncRNA features by Pyfeat

Feature name	Number of features
zCurve	3
gcContent	1
ATGC ratio	1
Cumulative Skew	2
Chou’s Pseudocomposition	84
monoMonoKGap	16
monoDiKGap	256
monoTriKGap	64
diMonoKGap	64
diDiKGap	1024
diTriKGap	256
triMonoKGap	256
triDiKGap	1024

#### Feature extraction of proteins

BioProt [39] utilizes various information to represent a protein. In this study, we use BioProt to obtain protein features and represent each protein as a 9,890-dimensional vector. The details are shown in Table 3.

**Table 3:** The protein features by BioProt

Feature group	Features	Number
Amino acid composition	Amino acid composition	20
	Dipeptide composition	400
	Tripeptide composition	8000
Autocorrelation	Normalized Moreau–Broto	240
	autocorrelation	
	Moran autocorrelation	240
	Geary autocorrelation	240
CTD	Composition	21
	Transition	21
	Distribution	105
Conjoint triad	Conjoint triad features	343
Quasi-sequence order	Sequence order coupling	60
	number	
	Quasi-sequence order	100
	descriptors	
Pseudo amino acid composition	Pseudo amino acid	50
	composition	
	Amphiphilic pseudo	50
	amino acid composition	

### Dimension reduction

The feature dimensions of lncRNAs and protein are reduced based on PCA, respectively. Two *d*-dimensional feature vectors are obtained and concatenated as a 2*d*-dimensional vector $\varvec{x}$ applied to represent an lncRNA–protein pair.

### LPI prediction framework

#### Problem description

For a given LPI dataset $D=(\varvec{X},\varvec{Y})$, where $(\varvec{x},\varvec{y})$ represents an lncRNA–protein pair (a training example), $\varvec{x}\in \varvec{X}$ denotes a 2*d*-dimensional LPI feature vector and $\varvec{y}\in \varvec{Y}$ denotes its label, we aim to classify unknown lncRNA–protein pairs.

For a feed-forward deep architecture with one original input layer, one output layer and (*m*-1) intermediate layers, suppose that $\varvec{o}_i$ ($i\in \{0,1,2, \cdots , m\}$) denotes the output in the *i*-th layer. For an lncRNA–protein pair $(\varvec{x}, \varvec{y})$, we want to learn a mapping ${F_i}$ based on GBDT to minimize the empirical loss *L* between the desired output $\varvec{y}$ and the final real output $\varvec{o}_m$ on the training data.

#### Gradient boosting decision trees

GBDT can generate highly robust, interpretable and competitive classification procedures, especially for exploiting less than clean data [29, 40, 41]. For an lncRNA–protein pair $(\varvec{x},\varvec{y})$, an estimator $f(\varvec{x})$ denotes an approximate function response to the label $\varvec{y}$, the GBDT model iteratively builds *K* different individual decision tree $\{g(\varvec{x}; \alpha _1), \dots , g(\varvec{x};\alpha _K)\}$ using the training data $D=(\varvec{X},\varvec{Y})$. And $f(\varvec{x})$ can be denoted as an expansion of individual decision tree $g(\varvec{x};\alpha _k)$ by Eq. (2).

2 $$\begin{aligned} \left\{ {\begin{array}{*{20}{l}} {f(\varvec{x}) = \sum \limits _{k = 1}^K {{f_k}(\varvec{x}) = \sum \limits _{k = 1}^K {{\beta _k}g(\varvec{x};{\alpha _k}} )} }\\ {g(\varvec{x};{\alpha _k}) = \sum \limits _{j = 1}^J {{\gamma _{ik}}I(\varvec{x} \in } {R_{ik}})} \end{array}} \right. \end{aligned}$$

where each tree splits the input space into *N* disjoint regions $\{R_{1k},\cdots , R_{jk}\}$ and calculates a constant value $\gamma _{ik}$ for the region $R_{jk}$ where $I = 1\,\,if\,\,\varvec{x} \in {R_{jk}};\,I = 0,\,otherwise$. $f_k(\varvec{x})$ denotes an addition function combined from the first decision tree to the *k*-th decision tree. The parameters $\alpha _{k}$ denotes the mean values of partition locations and the terminal leaf nodes for each partitioning variables in the *k*-th decision tree. The parameters $\beta _{k}$ denotes the weights used to determine how to effectively integrate the prediction results from individual decision trees when the leaf nodes of each collection are known. The two parameters $\alpha _{k}$ and $\beta _k$ can be estimated by minimizing a loss function $L(\varvec{y},f(\varvec{x}))$ by Eq. (3).

3 $$\begin{aligned} \begin{array}{l} ({\alpha _k},{\beta _k}) = \mathop {\arg \min }\limits _{\alpha ,\beta } \sum \limits _{i = 1}^N {L({\varvec{y}_i},\,\,{f_{k - 1}}({\varvec{x}_i}) + \beta g({\varvec{x}_i};\alpha ))} \\ \qquad \quad \,\,\,\, = \mathop {\arg \min }\limits _{\alpha ,\beta } \sum \limits _{i = 1}^N {L({\varvec{y}_i},\,\,{f_{k - 1}}({\varvec{x}_i}) + \beta \sum \limits _{j = 1}^J {{\gamma _j}I({\varvec{x}_i} \in } {R_j}))} \end{array} \end{aligned}$$

and

4 $$\begin{aligned} \begin{array}{l} {f_k}(\varvec{x}) = {f_{k - 1}}(\varvec{x}) + {\beta _k}g(\varvec{x};{\alpha _k}) = {f_{k - 1}}(\varvec{x}) + {\beta _k}\sum \limits _{j = 1}^J {{\gamma _{jk}}I(\varvec{x} \in } {R_{jk}}) \end{array} \end{aligned}$$

To solve the model (3), Friedman [42] proposed a gradient boosting approach. First, the parameters $\alpha _m$ can be estimated based on least square error:

5 $$\begin{aligned} \begin{array}{l} {\alpha _k} = \mathop {\arg \min }\limits _{\alpha ,\beta } \sum \limits _{i = 1}^N {[{{{\tilde{y}}}_{ik}} - \beta g({\varvec{x}_i};} \alpha ){]^2} = \mathop {\arg \min }\limits _{\alpha ,\beta } \sum \limits _{i = 1}^N {[{{{\tilde{y}}}_{ik}} - \beta \sum \limits _{j = 1}^J {{\gamma _j}I({\varvec{x}_i} \in } {R_j})} {]^2} \end{array} \end{aligned}$$

where ${{{{\tilde{y}}}_{im}}}$ denotes the gradient and is defined by Eq. (6).

6 $$\begin{aligned} {{{\tilde{y}}}_{ik}} = - {\left[\frac{{\partial L({\varvec{y}_i},f({\varvec{x}_i}))}}{{\partial f({\varvec{x}_i})}}\right]_{f(\varvec{x}) = {f_{k - 1}}(\varvec{x})}} \end{aligned}$$

The parameters $\beta _k$ can be determined by Eq. (7).

7 $$\begin{aligned} \begin{array}{l} {\beta _k} = \mathop {\arg \min }\limits _\beta \sum \limits _{i = 1}^N {L({\varvec{y}_i},{f_{k - 1}}({\varvec{x}_i}) + \beta g({\varvec{x}_i};{\alpha _k}))} \\ \quad \,\,= \mathop {\arg \min }\limits _{\beta } \sum \limits _{i = 1}^N {L({\varvec{y}_i},{f_{k - 1}}({\varvec{x}_i}) + \beta \sum \limits _{j = 1}^J {{\gamma _{jk}}I({\varvec{x}_i} \in } {R_{jk}}))} \end{array} \end{aligned}$$

The estimator $f_k(\varvec{x})$ for the *k*-th regression tree can be updated by Eq. (8)

8 $$\begin{aligned} f_k(\varvec{x})=f_{k-1}(\varvec{x})+\beta _{k}g(\varvec{x},\alpha _k) \end{aligned}$$

The final estimator $f(\varvec{x})$ can be obtained by Eq. (9)

9 $$\begin{aligned} f(\varvec{x}) = \sum \limits _{k = 1}^K {{f_k}} (\varvec{x}) \end{aligned}$$

The gradient boosting approach calculates the optimal values of the parameters $\alpha _m$ via minimizing the least square function defined by Eq. (5). The parameters $\beta _m$ can be solved by Eqs. (5) and (7). And the GBDT algorithm is described as Algorithm 1. 
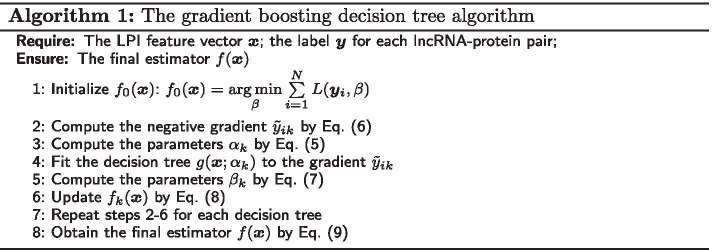


#### The multi-layered deep architecture with GBDT

We exploited a multi-layered deep architecture with GBDT to classify unknown lncRNA–protein pairs. Firstly, *m* gradient boosting decision trees are initialized. Initial forward mapping, inverse mapping, and output are then computed. Second, pseudo-label in the *m*-th layer is obtained based on the initialized output and real label. Third, the forward mapping for each regression tree is iteratively updated based on the computed pseudo-label at the last iteration. Fourth, the inverse mapping is iteratively learned based on the achieved forward mapping at the last iteration. Finally, the final label is output after *m* iterations.

**Phase I: Initialize GBDT**

It is very difficult to design a random tree structure based on the distribution from all potential tree configurations. Therefore, multiple Gaussian noise data are injected to the output in all intermediate layers. Given a deep structure with *m* layers, the initial forward mapping $F_i^{0}$ ($i\in \{1,2,..., m\}$) and the inverse mapping $G_i^{0}$ ($i\in \{2,3, ..., m\}$) are obtained by a few very tiny trees, where index 0 represents the tree structures achieved in the initialization procedure. In addition, the initial output $\varvec{o}_0$ is set as *X* and $\varvec{o}_i=F_i^{0}(\varvec{o}_{i-1})$ ($i\in \{1,2,..., m\}$).

The iterations are updated based on the learned forward mappings and inverse mappings. At each iteration *t*, we conduct Phases II-IV.

**Phase II: Compute the pseudo-label in the** *m* **-th layer**

The pseudo-label in the *m*-th layer can be computed based on the final output $\varvec{o}_m$ and the real label $\varvec{y}$, $\alpha$ is the learning rate by Eq. (10)

10 $$\begin{aligned} \varvec{p}_m^{t} = {\varvec{o}_m} - \alpha \frac{{\partial L({\varvec{o}_m},\varvec{y})}}{{\partial {\varvec{o}_m}}} \end{aligned}$$

**Phase III: Forward mapping**

At the *t*-th iteration, during the forward mapping, $F_i^{t}$ for each regression tree in a GBDT is first initialized by $F_i^{t}=F_i^{t-1}$ and updated based on a pseudo-labels $\varvec{p}_{i-1}^{t}$ with $\varvec{p}_{i-1}^{t}=G_i(\varvec{p}_i^{t})$. The details are described as follows.

For each regression tree in a GBDT, we define a reconstruction loss function as Eq. (11).

11 $$\begin{aligned} L_i^{forw}=||F_i^{t}(\varvec{o}_{i-1})-\varvec{p}_i^{t}|| \end{aligned}$$

The pseudo-residuals for each tree can be computed by Eq. (12).

12 $$\begin{aligned} {\varvec{r}_k^{forw}} = - \frac{{\partial {L_i^{forw}}}}{{\partial F_i^{t}({\varvec{o}_{i - 1}})}} \end{aligned}$$

When the pseudo-label in each layer is calculated, each $F_i^{t-1}$ can implement a gradient ascent towards its pseudo-residual by Eq. (12).

Each regression tree $g_k$ is fitted to $\varvec{r}_k^{forw}$ based on the training set ($\varvec{o}_{i-1},\varvec{r}_k^{forw}$) and the forward mapping $F_i^{t}$ for each tree can be updated by Eq. (13).

13 $$\begin{aligned} F_i^{t}=F_i^{t}+\gamma g_k \end{aligned}$$

Finally, we obtain the output for each layer by the forward mapping by Eq. (14).

14 $$\begin{aligned} \varvec{o}_i=F_i^{t}(\varvec{o}_{i-1}) \end{aligned}$$

The forward mapping procedures are described as Algorithm 2. 
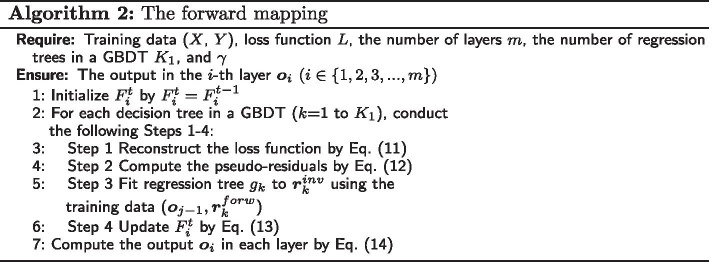


In this phase, we use a bottom up update technique, that is, $F_i$ will be updated before $F_j$ when $i<j$. In addition, each $F_i$ can run multiple rounds of additive boosting operations towards its current pseudo-label.

**Phase IV: Inverse mapping**

At the *t*-th iteration, for each decision tree, given the forward mapping $F_i^{t - 1}$ learned from the (*t*-2)-th iteration, we intend to achieve an “pseudo-inverse” mapping $G_i^{t}$ paired with each $F_i^{t - 1}$ satisfying $G_i^t(F_i^{t - 1}({\varvec{o}_{i - 1}})) \approx {\varvec{o}_{i - 1}}$ based on the following expected value of the reconstructed loss function by Eq. (15):

15 $$\begin{aligned} {\hat{G}}_i^{t} = \mathop {\arg \min }\limits _{G_i^{t}} {\mathrm{E}_x}[{L_i^{inv}}({\varvec{o}_{i - 1}},G_i^{t}(F_i^{t - 1}({\varvec{o}_{i - 1}})))] \end{aligned}$$

where $L_i^{inv}$ denotes the reconstructed loss in the *i*-th layer.

To build a more robust and generative model, random noises $\sigma$ are injected into the output in all intermediate layers:

16 $$\begin{aligned} \varvec{o}_{i-1}^{noise}=\varvec{o}_{i-1}^{noise}+\epsilon , \epsilon \sim \mathrm{N}(\varvec{0},diag({\sigma ^2})) \end{aligned}$$

For each regression tree $g_k$ in a GBDT, the reconstructed error can be computed by Eq. (17):

17 $$\begin{aligned} {L_i^{inv}} = ||G_i^t(F_i^{t - 1}({\varvec{o}_{i - 1}^{noise}} )) - ({\varvec{o}_{i - 1}^{noise}} )|| \end{aligned}$$

Based on the noise injection, each $G_i^{t-1}$ follows a gradient ascent towards the pseudo-residuals by Eq. (18)

18 $$\begin{aligned} {\varvec{r}_k^{inv}} = - \frac{{\partial L_i^{inv}}}{{\partial G_i^t(F_i^{t - 1}(\varvec{o}_{i - 1}^{noise}))}} \end{aligned}$$

where ${\varvec{r}_k^{inv}}$ denotes the pseudo-residuals of the *k*-th regression tree during the inverse mapping. For each regression tree $g_k$ in GBDT, we fit it to $\varvec{r}_k$ via the training set $(F_i^{t-1}(\varvec{o}_{j-1}^{noise}),\varvec{r}_k^{inv})$ and then update $G_i^{t}$ by Eq. (19).

19 $$\begin{aligned} G_i^{t}=G_i^{t}+\gamma g_k \end{aligned}$$

Finally, the pseudo-label in each intermediate layer can be propagated from the final layer to the first layer by Eq. (20):

20 $$\begin{aligned} \varvec{p}_{i-1}^{t}=G_i^{t}(\varvec{p}_i^{t}) \end{aligned}$$

For all intermediate layers and the final output layer ($i \in \{m,m-1,...,2\})$, the inverse mapping procedures are described as Algorithm 3. 
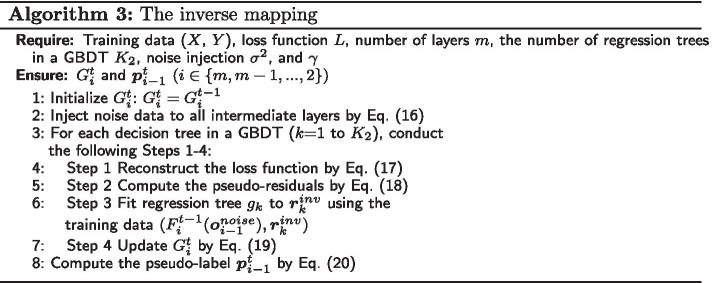


We can obtain the inverse mapping $G_i^{t}$ for the final output layer and all intermediate layers and the pseudo-labels $\varvec{p}_{i}^{t}$ for the first layer and all the intermediate layers. After finishing the *t*-th iteration, we continue the $(t+1)$-th iteration to update $F_i$ and $G_i$.

During LPI prediction, a linear classifier $\varvec{Y}=\varvec{XW}^{T}+b$ is applied to the forward mapping in the *m*-th layer. There are two main advantages. First, the *m*-1 layers can re-represent the LPI features as linearly separable as possible. Second, the corresponding inverse mapping in the *m*-th layer does not have to be computed because the pseudo-label in the (*m*-1)-th layer can be obtained based on the gradient of global loss related to the output in the (*m*-2)-th layer.

## Results

The experiments is mainly explored to empirically examine if the proposed LPI-deepGBDT method can effectively predict new LPIs.

### Evaluation metrics

The six measurements are utilized to evaluate the performance of LPI-deepGBDT: precision, recall, accuracy, F1-score, AUC and AUPR. For the six evaluation criteria, higher values depict better performance [43]. The experiments are repeatedly implemented for 20 times. The average performance for the 20 rounds is taken as the final performance. The six measurements are defined by Eqs. (21)–(24).

21 $$\begin{aligned} Precision= & {} \frac{TP}{TP+FP} \end{aligned}$$

22 $$\begin{aligned} Recall= & {} \frac{TP}{TP+FN} \end{aligned}$$

23 $$\begin{aligned} Accuracy= & {} \frac{TP +TN}{TN +FN +TP +FP} \end{aligned}$$

24 $$\begin{aligned} F1 - Score= & {} \frac{2TP}{2TP+FP+FN} \end{aligned}$$

where TP, TN, FP, and FN represent true positives, true negatives, false positives, and false negatives, respectively. Precision denotes the ratio of correctly predicted positive samples among all predicted positive samples. Recall represents the ratio of correctly predicted positive samples among all real positive samples. Accuracy denotes the ratio of correctly predicted positive and negative samples among all samples. F1-Score is harmonic mean between precision and recall. Area Under receiver operating Characteristic Curve (AUC) is used to measure the trade-off between TP ratio and FP ratio. Area Under Precision-Recall curve (AUPR) is applied to evaluate the trade-off between precision and recall.

### Experimental settings

The parameters in Pyfeat are set as: kgap=5, ktuple=3, optimum=1, pseudo=1, zcurve=1, gc=1, skew=1, atgc=1, monoMono=1, monoDi=1, monoTri=1, diMono=1, diDi=1, diTri=1, triMono=1, and triDi=1. All parameters in BioProt and LPI-SKF are the corresponding values provided by refs. [39] and [21], respectively. The deep GBDT architecture we used is (input-16-16-output). The parameters in the remaining methods are set the values when the corresponding methods obtain the best performance. The details are described in Table 4.

**Table 4:** Parameter settings

Method	Parameter setting
LPI-BLS	s = 1, c = 10**-10, N1 = 3, N2 = 60, N3 = 900
LPI-CastBoost	learning_rate = 0.5, loss_function = ‘Logloss’
	logging_level = ’Verbose’
PLIPCOM	learning_rate = 0.01, n_estimators = 100
	min_samples_split = 2, max_depth = 3
LPI-deepGBDT	target_lr = 1.0, epsilon = 0.3, n_rounds=3, d = 100
	max_depth = 5, num_boost_round = 5, n_epochs = 15

Therefore, we select two 100-dimensional vectors to represent lncRNA and protein, respectively. Three 5-fold Cross Validations (CVs) are carried out to evaluate the performance of LPI-deepGBDT.

5-fold CV on lncRNAs (CV1): 80% of lncRNAs are extracted as train set and the remaining is test set in each round.

5-fold CV on proteins (CV2): 80% of proteins are extracted as train set and the remaining is test set in each round.

5-fold CV on lncRNA–protein pairs (CV3): 80% of lncRNA–protein pairs are extracted as train set and the remaining is test set in each round.

The three CVs refer to potential LPI identification for (1) a new (unknown) lncRNA without interaction information, (2) a new protein without interaction information, and (3) lncRNA–protein pairs, respectively.

### Comparison with five state-of-the-art LPI prediction methods

We compare the proposed LPI-deepGBDT framework with five classical LPI identification models to measure the classification performance and robustness of LPI-deepGBDT, that is, LPI-BLS, LPI-CatBoost, PLIPCOM, LPI-SKF and LPI-HNM. The number of negative samples is set as the same as positive samples. The best performance is illustrated in boldface in each row in Tables 5, 6 and 7.

Table 5 gives the comparative results of the five LPI identification models in terms of the six measurements under CV1. It can be observed that LPI-deepGBDT achieves better average recall, accuracy, F1-score, AUC and AUPR than LPI-BLS, LPI-CatBoost, PLIPCOM, and LPI-HNM on five LPI datasets. For example, LPI-deepGBDT obtains the best average F1-score value of 0.7586, 8.99%, 9.83%, 1.61%, 22.70% and 8.37% superior than LPI-BLS, LPI-CatBoost, PLIPCOM, LPI-SKF, and LPI-HNM, respectively. More importantly, it calculates the best AUC value of 0.8321, 1.63%, 8.32%, 2.37%, 0.02% and 6.26% better than the above five models, respectively. It also achieves the best average AUPR of 0.8095, 1.85%, 5.53%, 0.77%, 0.02% and 0.24% higher than the five methods, respectively.

LPI-BLS, LPI-CatBoost, PLIPCOM and LPI-HNM are four state-of-the-art supervised learning-based LPI prediction methods and LPI-deepGBDT computes better performance than them. The results suggest the powerful classification ability of LPI-deepGBDT under CV1. More importantly, although LPI-deepGBDT computes slightly lower precision than LPI-SKF, other five measurements are better than LPI-SKF. LPI-SKF is one network-based LPI inference algorithm. The type of methods have one limitation, that is, they can not be applied to predict possible interaction information for an orphan lncRNA. Therefore, LPI-deepGBDT is appropriate to prioritize underlying proteins associated with a new lncRNA.

**Table 5:** The performance of five LPI prediction methods on CV1

Metric	Dataset	LPI-BLS	LPI-CatBoost	PLIPCOM	LPI-SKF	LPI-HNM	LPI-deepGBDT
Precision	Dataset 1	0.8458 ± 0.0014	0.8317 ± 0.0132	0.8428 ± 0.0060	**0.8757** ± **0.0086**	0.7006 ± 0.0171	0.8457 ± 0.0046
	Dataset 2	0.8547 ± 0.0031	0.8220 ± 0.0139	0.8537 ± 0.0065	**0.8627** ± **0.0223**	0.7009 ± 0.0169	0.8567 ± 0.0038
	Dataset 3	0.7110 ± 0.0011	0.6871 ± 0.0060	0.7173 ± 0.0084	**0.7298** ± **0.0153**	0.7054 ± 0.0169	0.7089 ± 0.0115
	Dataset 4	0.5653 ± 0.0088	0.4613 ± 0.0369	0.4894 ± 0.0508	0.6108 ± 0.0249	**0.6624** ± **0.0501**	0.5870 ± 0.0289
	Dataset 5	0.7901 ± 0.0021	0.7713 ± 0.0040	0.7721 ± 0.0021	0.7517 ± 0.0098	0.7959 ± 0.0157	**0.8018** ± **0.0189**
	Ave.	0.7534	0.7147	0.7351	**0.7661**	0.7130	0.7600
Recall	Dataset 1	0.6550 ± 0.0009	0.8331 ± 0.0140	**0.9632** ± **0.0028**	0.5932 ± 0.0156	0.7134 ± 0.0152	0.9456 ± 0.0070
	Dataset 2	0.6738 ± 0.0013	0.8399 ± 0.0201	**0.9628** ± **0.0043**	0.5212 ± 0.0107	0.6893 ± 0.0146	0.9495 ± 0.0063
	Dataset 3	0.6270 ± 0.0006	0.6154 ± 0.0241	0.7618 ± 0.0141	0.6226 ± 0.0058	0.6930 ± 0.0113	**0.7649** ± **0.0249**
	Dataset 4	0.5328 ± 0.0074	0.3539 ± 0.0700	0.3190 ± 0.0668	0.6056 ± 0.0280	**0.6342** ± **0.0396**	0.3613 ± 0.0453
	Dataset 5	0.7063 ± 0.0038	0.7921 ± 0.0135	**0.8569** ± **0.0037**	0.6727 ± 0.0037	0.6682 ± 0.0077	0.8425 ± 0.0261
	Ave.	0.6390	0.6869	0.7727	0.6030	0.6796	**0.7728**
Accuracy	Dataset 1	0.7512 ± 0.0005	0.8310 ± 0.0071	0.8917 ± 0.0039	0.7254 ± 0.0032	0.6571 ± 0.0112	**0.8964** ± **0.0032**
	Dataset 2	0.7620 ± 0.0018	0.8258 ± 0.0064	**0.8987** ± **0.0034**	0.7065 ± 0.0081	0.6474 ± 0.0088	0.8952 ± 0.0024
	Dataset 3	0.6605 ± 0.0012	0.6677 ± 0.0091	**0.7298** ± **0.0034**	0.6544 ± 0.0092	0.6585 ± 0.0097	0.7236 ± 0.0043
	Dataset 4	0.5424 ± 0.0048	0.4801 ± 0.0201	0.4972 ± 0.0306	0.5727 ± 0.0196	**0.6100** ± **0.0274**	0.5506 ± 0.0167
	Dataset 5	0.7337 ± 0.0025	0.7785 ± 0.0067	0.8018 ± 0.0018	0.6726 ± 0.0036	0.7117 ± 0.0053	**0.8129** ± **0.0132**
	Ave.	0.6900	0.7166	0.7638	0.6663	0.6569	**0.7757**
F1-score	Dataset 1	0.7381 ± 0.0012	0.8314 ± 0.0067	**0.8989** ± **0.0033**	0.6298 ± 0.0070	0.7069 ± 0.0148	0.8927 ± 0.0031
	Dataset 2	0.7533 ± 0.0020	0.8282 ± 0.0067	0.9048 ± 0.0027	0.5828 ± 0.0117	0.6949 ± 0.0140	**0.9105** ± **0.0024**
	Dataset 3	0.6663 ± 0.0008	0.6480 ± 0.0148	**0.7377** ± **0.0034**	0.5950 ± 0.0086	0.6991 ± 0.0119	0.7337 ± 0.0068
	Dataset 4	0.5483 ± 0.0081	0.3812 ± 0.0573	0.3783 ± 0.0597	0.5401 ± 0.0232	**0.6480** ± **0.0445**	0.4397 ± 0.0362
	Dataset 5	0.7458 ± 0.0030	0.7812 ± 0.0080	0.8121 ± 0.0018	0.6345 ± 0.0041	0.7264 ± 0.0061	**0.8165** ± **0.0134**
	Ave.	0.6904	0.6940	0.7464	0.5964	0.6951	**0.7586**
AUC	Dataset 1	0.9192 ± 0.0005	0.8860 ± 0.0048	0.9313 ± 0.0030	0.9344 ± 0.0073	0.7774 ± 0.0147	**0.9346** ± **0.0040**
	Dataset 2	0.9301 ± 0.0017	0.8909 ± 0.0044	0.9389 ± 0.0034	0.9199 ± 0.0149	0.7677 ± 0.0133	**0.9398** ± **0.0028**
	Dataset 3	0.7849 ± 0.0020	0.7151 ± 0.0112	**0.8223** ± **0.0029**	0.8117 ± 0.0159	0.7794 ± 0.0126	0.8083 ± 0.0042
	Dataset 4	0.5843 ± 0.0094	0.4726 ± 0.0270	0.4891 ± 0.0326	0.6479 ± 0.0379	**0.7038** ± **0.0438**	0.5790 ± 0.0207
	Dataset 5	0.8738 ± 0.0028	0.8498 ± 0.0064	0.8806 ± 0.0019	0.8455 ± 0.0076	0.8718 ± 0.0074	**0.8988** ± **0.0126**
	Ave.	0.8185	0.7629	0.8124	0.8319	0.7800	**0.8321**
AUPR	Dataset 1	0.8851 ± 0.0022	0.8936 ± 0.0049	**0.9224** ± **0.0037**	0.9196 ± 0.0092	0.8260 ± 0.0180	0.8889 ± 0.0091
	Dataset 2	0.8975 ± 0.0032	0.8929 ± 0.0050	**0.9266** ± **0.0044**	0.8787 ± 0.0260	0.8039 ± 0.0187	0.8991 ± 0.0068
	Dataset 3	0.7469 ± 0.0006	0.7024 ± 0.0109	**0.8060** ± **0.0044**	0.7772 ± 0.0198	0.8039 ± 0.0161	0.7792 ± 0.0070
	Dataset 4	0.5851 ± 0.0109	0.5074 ± 0.0254	0.4987 ± 0.0272	0.6348 ± 0.0340	**0.7435** ± **0.0689**	0.5965 ± 0.0176
	Dataset 5	0.8579 ± 0.0036	0.8274 ± 0.0079	0.8626 ± 0.0027	0.8364 ± 0.0170	0.8601 ± 0.0118	**0.8837** ± **0.0121**
	Ave.	0.7945	0.7647	0.8033	0.8093	0.8075	**0.8095**

Table 6 depicts the performance of LPI-BLS, LPI-CatBoost, PLIPCOM, LPI-SKF, LPI-HNM, and LPI-deepGBDT under CV2. The results show that the performance of LPI-deepGBDT is slightly lower than LPI-HNM. Under CV2, 80% proteins are extracted as training set and the remaining is test set in each round. That is, there will be relatively higher proteins for which association information is masked, thereby resulting in the reduction of samples and affecting the performance of LPI-deepGBDT. Compared to other five methods, LPI-HNM may be relatively robust to data abundant level when predicting possible lncRNAs for a new protein.

More importantly, LPI-deepGBDT computes the best average AUC and AUPR in comparing to LPI-BLS, LPI-CatBoost, and PLIPCOM. For example, LPI-deepGBDT obtains the best average AUC of 0.6815, 21.97%, 9.24%, and 4.01% superior than LPI-BLS, LPI-CatBoost, and PLIPCOM, respectively. LPI-deepGBDT achieves the best average AUPR of 0.6771, 15.74%, 10.37%, and 6.78% better than the above three methods, respectively. AUC and AUPR are two more important evaluation criteria compared to other four measurements. LPI-deepGBDT outperforms LPI-BLS, LPI-CatBoost, and PLIPCOM in terms of AUC and AUPR. The results suggest that LPI-deepGBDT is one appropriate LPI prediction algorithm.

In particular, LPI-BLS is an ensemble learning-based model. LPI-deepGBDT significantly outperforms LPI-BLS based on AUC and AUPR. The results illustrate that LPI-deepGBDT may obtain better ensemble performance. In addition, LPI-CatBoost and PLIPCOM are two categorical boosting techniques. LPI-deepGBDT, integrating the idea of deep architecture, obtains better performance than the two methods. It shows that deep learning may more effectively learn the relevances between lncRNAs and proteins. Although LPI-SKF computes better AUPR than LPI-deepGBDT, LPI-SKF is a network-based model. Network-based methods can not reveal association information for an orphan protein. In summary, LPI-deepGBDT may be applied to infer possible interacting lncRNAs for a new protein.

**Table 6:** The performance of five LPI prediction methods on CV2

Metric	Dataset	LPI-BLS	LPI-CatBoost	PLIPCOM	LPI-SKF	LPI-HNM	LPI-deepGBDT
Precision	Dataset 1	0.5370 ± 0.0347	0.3405 ± 0.1562	0.3541 ± 0.1209	**0.7009** ± **0.1208**	0.6836 ± 0.1148	0.4413 ± 0.1452
	Dataset 2	0.5769 ± 0.0287	0.3468 ± 0.1536	0.3879 ± 0.1793	0.6138 ± 0.1316	**0.6227** ± **0.1840**	0.6190 ± 0.0982
	Dataset 3	0.4479 ± 0.0234	0.5419 ± 0.0476	0.3772 ± 0.1050	0.6639 ± 0.1119	**0.6842** ± **0.0844**	0.5312 ± 0.0742
	Dataset 4	0.5319 ± 0.0042	0.6023 ± 0.0286	0.7413 ± 0.0151	0.7261 ± 0.0412	0.6635 ± 0.0230	**0.7421** ± **0.0133**
	Dataset 5	0.4164 ± 0.0122	**0.7868** ± **0.0085**	0.7459 ± 0.0037	0.7264 ± 0.1465	0.7700 ± 0.0505	0.7658 ± 0.0349
	Ave.	0.5020	0.5237	0.5213	**0.6862**	0.6848	0.6199
Recall	Dataset 1	0.5264 ± 0.0130	0.2567 ± 0.1423	0.2165 ± 0.0725	0.5415 ± 0.0702	**0.7060** ± **0.0876**	0.2298 ± 0.1220
	Dataset 2	0.5486 ± 0.0204	0.2325 ± 0.1309	0.1744 ± 0.1197	0.4114 ± 0.0551	**0.6568** ± **0.1041**	0.2067 ± 0.0915
	Dataset 3	0.4819 ± 0.0104	0.3637 ± 0.0817	0.3023 ± 0.1209	0.4982 ± 0.0746	**0.6651** ± **0.0211**	0.3525 ± 0.1286
	Dataset 4	0.5479 ± 0.0042	0.5278 ± 0.0600	0.6730 ± 0.0125	0.5402 ± 0.0415	0.6411 ± 0.0329	**0.6978** ± **0.0273**
	Dataset 5	0.7993 ± 0.0470	0.8122 ± 0.0338	0.8473 ± 0.0155	0.5811 ± 0.0589	0.7394 ± 0.0156	**0.8684** ± **0.0565**
	Ave.	0.5808	0.4386	0.4427	0.5145	**0.6817**	0.4710
Accuracy	Dataset 1	0.5382 ± 0.0252	0.5204 ± 0.0694	0.5173 ± 0.0424	0.5867 ± 0.0757	**0.6518** ± **0.0350**	0.5386 ± 0.0615
	Dataset 2	0.5672 ± 0.0181	0.5092 ± 0.0641	0.5298 ± 0.0562	0.5220 ± 0.0482	**0.6474** ± **0.0736**	0.5609 ± 0.0430
	Dataset 3	0.4708 ± 0.0139	0.5361 ± 0.0321	0.4899 ± 0.0349	0.5584 ± 0.0777	**0.6347** ± **0.0312**	0.5284 ± 0.0409
	Dataset 4	0.5135 ± 0.0038	0.5767 ± 0.0126	0.7172 ± 0.0109	0.6202 ± 0.0332	0.6150 ± 0.0286	**0.7261** ± **0.0104**
	Dataset 5	0.5089 ± 0.0004	0.7951 ± 0.0141	0.7785 ± 0.0051	0.6636 ± 0.0644	0.7117 ± 0.0144	**0.7985** ± **0.0117**
	Ave.	0.5197	0.5875	0.6065	0.5902	**0.6521**	0.6305
F1-score	Dataset 1	0.5285 ± 0.0228	0.2567 ± 0.1423	0.2494 ± 0.0853	0.5399 ± 0.0745	**0.6818** ± **0.0428**	0.2697 ± 0.1242
	Dataset 2	0.5617 ± 0.0246	0.2622 ± 0.1347	0.2131 ± 0.1301	0.4092 ± 0.0634	**0.6295** ± **0.1274**	0.2629 ± 0.1012
	Dataset 3	0.4635 ± 0.0172	0.4175 ± 0.0750	0.3144 ± 0.1120	0.4929 ± 0.0804	**0.6719** ± **0.0487**	0.3791 ± 0.0995
	Dataset 4	0.5372 ± 0.0005	0.5389 ± 0.0305	0.7030 ± 0.0103	0.5468 ± 0.0408	0.6521 ± 0.0280	**0.7160** ± **0.0142**
	Dataset 5	0.5467 ± 0.0250	0.7970 ± 0.0184	0.7920 ± 0.0071	0.5908 ± 0.0734	0.7537 ± 0.0290	**0.8115** ± **0.0084**
	Ave.	0.5275	0.4545	0.4544	0.5159	**0.6778**	0.4878
AUC	Dataset 1	0.5701 ± 0.0508	0.5659 ± 0.0734	0.5397 ± 0.0855	0.6293 ± 0.1142	**0.8013** ± **0.0902**	0.5419 ± 0.0863
	Dataset 2	0.6227 ± 0.0328	0.5173 ± 0.0987	0.5895 ± 0.0743	0.5235 ± 0.0899	**0.7578** ± **0.1278**	0.6347 ± 0.0798
	Dataset 3	0.4443 ± 0.0269	0.5373 ± 0.0421	0.5084 ± 0.0512	0.5848 ± 0.1577	**0.7595** ± **0.0402**	0.5625 ± 0.0508
	Dataset 4	0.5206 ± 0.0088	0.6004 ± 0.0148	0.7791 ± 0.0124	0.7202 ± 0.0571	0.7134 ± 0.0528	**0.7883** ± **0.0115**
	Dataset 5	0.5013 ± 0.0025	0.8717 ± 0.0133	0.8544 ± 0.0063	0.8000 ± 0.1136	**0.8959** ± **0.0212**	0.8802 ± 0.0172
	Ave.	0.5318	0.6185	0.6542	0.6516	**0.7856**	0.6815
AUPR	Dataset 1	0.5429 ± 0.0415	0.5303 ± 0.0744	0.5099 ± 0.0686	0.7347 ± 0.1155	**0.8520** ± **0.0714**	0.5539 ± 0.0754
	Dataset 2	0.5672 ± 0.0181	0.4973 ± 0.0760	0.5299 ± 0.0719	0.5965 ± 0.1215	**0.7137** ± **0.2185**	0.6272 ± 0.0669
	Dataset 3	0.4600 ± 0.0243	0.5438 ± 0.0333	0.5197 ± 0.0420	0.6556 ± 0.1277	**0.7782** ± **0.0554**	0.5614 ± 0.0422
	Dataset 4	0.5525 ± 0.0034	0.6161 ± 0.0211	0.7778 ± 0.0168	0.7415 ± 0.0543	0.7491 ± 0.0348	**0.7788** ± **0.0151**
	Dataset 5	0.7308 ± 0.0046	0.8471 ± 0.0164	0.8187 ± 0.0119	0.7600 ± 0.1657	**0.8836** ± **0.0563**	0.8643 ± 0.0253
	Ave.	0.5707	0.6069	0.6312	0.6977	**0.7953**	0.6771

The experimental results under CV3 are shown in Table 7. The comparative results demonstrate that LPI-deepGBDT computed the best average precision, recall, accuracy, F1-score, AUC, and AUPR over all datasets. For example, LPI-deepGBDT obtains the best average F1-score value of 0.8429, 14.83%, 10.77%, 3.10%, 16.73% and 18.43% superior than LPI-BLS, LPI-CatBoost, PLIPCOM, LPI-SKF and LPI-HNM, respectively. More importantly, it calculates the best AUC value of 0.9073, 4.93%, 11.21%, 3.32%, 0.12% and 14.49%, better than the above five models, respectively. It also achieves the best average AUPR of 0.8849, 5.82%, 8.84%, 2.59%, 2.62% and 9.13% higher than the five methods, respectively. The results characterize the superior classification performance of LPI-deepGBDT. Therefore, LPI-deepGBDT can precisely discover the potential relationships between lncRNAs and proteins based on known association information.

In addition, we investigate the performance computed by all six LPI prediction methods under the three different cross validations. The results from Tables 5, 6 and 7 show that LPI-BLS, LPI-CatBoost, PLIPCOM, LPI-SKF, and LPI-deepGBDT achieve much better performance under CV3 than CV1, followed by CV2, regardless of precision, recall, accuracy, F1-score, AUC or AUPR. Under CV3, cross validations are conducted on all lncRNA–protein pairs and 80% lncRNA–protein pairs are used to train the model and the remaining 20% lncRNA–protein pairs are applied to test the model. However, under CV1 or CV2, cross validations are implemented on lncRNAs or proteins, that is, 80% lncRNAs or proteins are applied to train the model and the remaining 20% lncRNAs or proteins are used to test the model. CV3 may provide more LPI information relative to CV1 and CV2. The result suggest that abundant data contribute to improve the prediction performance of LPI identification models.

**Table 7:** The performance of five LPI prediction methods on CV3

Metric	Dataset	LPI-BLS	LPI-CatBoost	PLIPCOM	LPI-SKF	LPI-HNM	LPI-deepGBDT
Precision	Dataset 1	0.8539 ± 0.0012	0.8340 ± 0.0170	0.8440 ± 0.0045	0.7979 ± 0.0337	0.7192 ± 0.0076	**0.8572 ± 0.0143**
	Dataset 2	**0.8668 ± 0.0018**	0.8191 ± 0.0224	0.8478 ± 0.0021	0.7902 ± 0.0059	0.7104 ± 0.0081	0.8638 ± 0.0089
	Dataset 3	0.7142 ± 0.0005	0.7349 ± 0.0183	0.7182 ± 0.0138	**0.7631 ± 0.0095**	0.7052 ± 0.0055	0.7565 ± 0.0313
	Dataset 4	0.7012 ± 0.0065	0.6289 ± 0.0277	0.7498 ± 0.0144	0.7948 ± 0.0070	0.6527 ± 0.0124	**0.8085 ± 0.0230**
	Dataset 5	0.7971 ± 0.0031	0.7425 ± 0.0047	0.7761 ± 0.0016	0.8248 ± 0.0011	0.8069 ± 0.0032	**0.8578 ± 0.0066**
	Ave.	0.7866	0.7518	0.7872	0.7942	0.7189	**0.8287**
Recall	Dataset 1	0.6565 ± 0.0083	0.8308 ± 0.0154	0.9652 ± 0.0080	0.9379 ± 0.0283	0.6811 ± 0.0043	**0.9684 ± 0.0071**
	Dataset 2	0.6603 ± 0.0068	0.8451 ± 0.0242	0.9504 ± 0.0012	0.6910 ± 0.0092	0.6485 ± 0.0116	**0.9611 ± 0.0137**
	Dataset 3	0.6313 ± 0.0075	0.6951 ± 0.0336	**0.7612 ± 0.0237**	0.6745 ± 0.0065	0.6712 ± 0.0062	0.7588 ± 0.0939
	Dataset 4	0.6445 ± 0.0046	0.5863 ± 0.0638	0.6988 ± 0.0143	0.7007 ± 0.0052	0.6177 ± 0.0162	**0.7903 ± 0.0650**
	Dataset 5	0.7194 ± 0.0014	0.8691 ± 0.0035	0.8659 ± 0.0030	0.7304 ± 0.0006	0.6787 ± 0.0025	**0.9003 ± 0.0151**
	Ave.	0.6624	0.7652	0.8483	0.7469	0.6594	**0.8745**
Accuracy	Dataset 1	0.7604 ± 0.0027	0.8319 ± 0.0170	**0.8933 ± 0.0020**	0.8488 ± 0.0136	0.6521 ± 0.0067	0.8877 ± 0.0075
	Dataset 2	0.7687 ± 0.0032	0.8264 ± 0.0107	0.8976 ± 0.0018	0.6965 ± 0.0057	0.6439 ± 0.0087	**0.9570 ± 0.0125**
	Dataset 3	0.6635 ± 0.0038	0.7194 ± 0.0061	0.7302 ± 0.0044	0.6745 ± 0.0065	0.6462 ± 0.0048	**0.7683 ± 0.0136**
	Dataset 4	0.6542 ± 0.0044	0.6095 ± 0.0138	0.7322 ± 0.0092	0.7007 ± 0.0052	0.5958 ± 0.0107	**0.8047 ± 0.0204**
	Dataset 5	0.7428 ± 0.0030	0.7837 ± 0.0030	0.8081 ± 0.0010	0.7304 ± 0.0006	0.7193 ± 0.0017	**0.9355 ± 0.0028**
	Ave.	0.7179	0.7542	0.8123	0.7302	0.6515	** 0.8583**
F1-score	Dataset 1	0.7421 ± 0.0048	0.8315 ± 0.0082	**0.9005 ± 0.0020**	0.8614 ± 0.0077	0.6996 ± 0.0055	0.8954 ± 0.0061
	Dataset 2	0.7495 ± 0.0051	0.8295 ± 0.0094	0.9044 ± 0.0016	0.6565 ± 0.0071	0.6780 ± 0.0093	**0.9200 ± 0.0101**
	Dataset 3	0.6702 ± 0.0019	0.7110 ± 0.0095	0.7379 ± 0.0043	0.6359 ± 0.0072	0.6878 ± 0.0045	**0.8269 ± 0.0297**
	Dataset 4	0.6716 ± 0.0054	0.5881 ± 0.0264	0.7226 ± 0.0091	0.6636 ± 0.0057	0.6347 ± 0.0142	**0.8042 ± 0.0306**
	Dataset 5	0.7563 ± 0.0022	0.8007 ± 0.0020	0.8186 ± 0.0011	0.6923 ± 0.0007	0.7373 ± 0.0015	**0.8784 ± 0.0041**
	Ave.	0.7179	0.7521	0.8168	0.7019	0.6875	**0.8429**
AUC	Dataset 1	0.9247 ± 0.0012	0.8846 ± 0.0060	0.9292 ± 0.0016	0.9293 ± 0.0120	0.7800 ± 0.0108	**0.9354 ± 0.0072**
	Dataset 2	0.9352 ± 0.0011	0.8918 ± 0.0055	0.9389 ± 0.0015	0.8893 ± 0.0136	0.7599 ± 0.0134	**0.9423 ± 0.0060**
	Dataset 3	0.7883 ± 0.6735	0.7940 ± 0.0049	0.8229 ± 0.0025	0.8493 ± 0.0130	0.7693 ± 0.0083	**0.8526 ± 0.0116**
	Dataset 4	0.7823 ± 0.0069	0.6421 ± 0.0122	0.8047 ± 0.0095	0.9024 ± 0.0105	0.6824 ± 0.0236	**0.8542 ± 0.0137**
	Dataset 5	0.8826 ± 0.0031	0.8156 ± 0.0020	0.8903 ± 0.0010	**0.9609 ± 0.0013**	0.8874 ± 0.0029	0.9523 ± 0.0012
	Ave.	0.8626	0.8056	0.8772	0.9062	0.7758	**0.9073**
AUPR	Dataset 1	0.8852 ± 0.0006	0.8904 ± 0.0084	0.9208 ± 0.0028	**0.9290 ± 0.0155**	0.8297 ± 0.0084	0.9043 ± 0.0162
	Dataset 2	0.9013 ± 0.0035	0.8926 ± 0.0049	0.9049 ± 0.0028	0.8956 ± 0.0128	0.7897 ± 0.0120	**0.9242 ± 0.0171**
	Dataset 3	0.7520 ± 0.0006	0.7936 ± 0.0062	0.8081 ± 0.0038	**0.8560 ± 0.0162**	0.7956 ± 0.0077	0.8016 ± 0.0190
	Dataset 4	0.7585 ± 0.0119	0.6629 ± 0.0190	0.8032 ± 0.0104	0.6683 ± 0.0061	0.7261 ± 0.0145	**0.8488 ± 0.0175**
	Dataset 5	0.8698 ± 0.0032	0.7943 ± 0.0019	0.8731 ± 0.0016	**0.9596 ± 0.0021**	0.8792 ± 0.0031	0.9457 ± 0.0033
	Ave.	0.8334	0.8067	0.8620	0.8617	0.8041	**0.8849**

### Case study

In this section, we aim to mine possible association data for a new lncRNA/protein or based on known LPIs.

#### Identifying potential proteins for a new lncRNA

RN7SL1 is an endogenous RNA. The lncRNA is usually protected by RNA-binding protein SRP9/14. Its increase can alter the stoichiometry with SRP9/14 and thus produce unshielded RN7SL1 in stromal exosomes. After exosome transfer to breast cancer cells, unshielded RN7SL1 can activate breast cancer RIG-I and promote tumor growth, metastasis, and therapy resistance [44]. Hepatocellular carcinoma patients with higher RN7SL1 concentrations also show lower survival rates. RN7SL1 may enhance hepatocellular carcinoma cell proliferation and clonogenic growth [45].

In this section, we mask all interaction information for RN7SL1 and want to infer possible proteins interacting with the lncRNA. The experiments are repeated for 10 times and the interaction probabilities between RN7SL1 and other proteins are averaged over the 10 time results. The predicted top 5 proteins interacting with RN7SL1 on human LPI datasets are described in Table 8. In Dataset 1, we can observe that RN7SL1 is predicted to interact with Q15465. Q15465 displays a cholesterol transferase and autoproteolysis activity in the reticulum endoplasmic. Its N-product is a morphogen required for diverse patterning events during development. It induces ventral cell fate in somites and the neural tubes. It is required for axon guidance and densely related to the anterior-posterior axis patterning in the developing limb bud [35]. In the dataset, RN7SL1 may associate with 59 proteins. In other two datasets, there does not exist any associated lncRNAs for Q15465. Although the interaction between RN7SL1 and Q15465 hasn’t been validated, among all possible associated 59 proteins, the protein is ranked as 4, 6, 8, 9, and 14 by LPI-CatBoost, PLIPCOM, LPI-SKF, LPI-HNM, and LPI-BLS, respectively. Therefore, the association between RN7SL1 and Q15465 need further validation.

In Dataset 2, we predict that Q13148, P07910, and Q9NZI8 may interact with RN7SL1. The interaction between Q9NZI8 and RN7SL1 is known in Dataset 3. Q13148 is a RNA-binding protein involved in various procedures in RNA biogenesis and processing. The protein controls the splicing in numerous non-coding and protein-coding RNAs, for example, proteins involved in neuronal survival and mRNAs encoding proteins related to neurodegenerative diseases. It plays important roles in maintaining mitochondrial homeostasis, mRNA stability and circadian clock periodicity, the normal skeletal muscle formation and regeneration. In Dataset 2, RN7SL1 may associate with 84 proteins. Among the 84 underlying proteins for RN7SL1, the rankings of Q13148 predicted by LPI-deepGBDT LPI-CatBoost, PLIPCOM, LPI-SKF, LPI-BLS, and LPI-HNM are 2, 3, 1, 3, 2, and 6, respectively. That is, all the six LPI identification models predict that there may be interaction between Q13148 and RN7SL1. Therefore, we infer that Q13148 may possibly interact with RN7SL1.

More importantly, in Dataset 2, P07910 binds to pre-mRNA and regulates the stability and translation level of bound mRNA molecules. The protein is involved in the early procedures of spliceosome assembly and pre-mRNA splicing. In other two human LPI datasets, there are no any known associated lncRNAs for P07910. Among 84 potential associated proteins for RN7SL1, P07910 is ranked as 3, 7, 8, 9, 11, and 9 by LPI-deepGBDT, LPI-BLS, LPI-CatBoost, PLIPCOM, LPI-SKF, and LPI-HNM, respectively. The ranking are relatively higher. Therefore, we predict that P07910 may associate with RN7SL1.

In Dataset 3, we observe that Q9UKV8 and Q9Y6M1 may interact with RN7SL1. The interactions between RN7SL1 and the two proteins can be retrieved in Dataset 1. That is, the predicted top 5 interaction data by LPI-deepGBDT can be validated by publications. In summary, the results from case analyses based on association prediction for a new lncRNA suggest that LPI-deepGBDT can be utilized to identify new proteins associated with a new lncRNA.

**Table 8:** The predicted top 5 proteins interacting with RN7SL1

Dataset	Proteins	Confirmed	LPI-deepGBDT	LPI-BLS	LPI-CatBoost	PLIPCOM	LPL-SKF	LPI-HNM
Dataset 1	O00425	YES	1	2	2	4	7	3
	Q9Y6M1	YES	2	8	3	8	6	2
	Q15465	NO	3	14	4	6	8	9
	Q15717	YES	4	1	1	2	21	4
	Q9UKV8	YES	5	4	7	14	1	8
Dataset 2	Q8IUX4	YES	1	6	2	2	8	5
	Q13148	NO	2	2	3	1	3	6
	P07910	NO	3	7	8	9	11	9
	Q9NZI8	NO	4	5	6	3	5	7
	Q9HCE1	YES	5	9	4	4	10	4
Dataset 3	Q9UKV8	NO	1	7	5	9	10	7
	Q9NUL5	YES	2	1	1	1	1	5
	Q9Y6M1	NO	3	4	4	5	6	6
	O00425	YES	4	3	3	2	3	1
	Q9NZI8	YES	5	6	2	3	2	2

#### Finding potential lncRNAs interacting with a new protein

Q9UL18 is a protein required by RNA-mediated gene silencing. The protein can repress the translation of mRNAs complementary to them by binding to short RNAs or short interfering RNAs. It lacks endonuclease activity and thus can cleave target mRNAs. It is still required by transcriptional gene silencing of promoter regions complementary to bound short antigene RNAs [35]. In this section, we mask the interaction information for Q9UL18 and intend to find associated lncRNAs for the protein. The predicted top 5 lncRNAs on three human LPI dataset are shown in Table 9.

In Datasets 1-3, Q9UL18 may interact with 935, 885, and 990 lncRNAs. It can be seen that all the predicted top 5 interactions on each dataset are validated as known LPIs. The results suggest that LPI-deepGBDT can be applied to prioritize possible lncRNAs for a new protein.

**Table 9:** The predicted top 5 lncRNAs interacting with Q9UL18

Dataset	lncRNAs	Confirmed	LPI-deepGBDT	LPI-BLS	LPI-CatBoost	PLIPCOM	LPL-SKF	LPI-HNM
Dataset 1	RPI001_1006774	YES	1	439	614	566	29	593
	RP11-4O1	YES	2	169	177	204	558	10
	LUCAT1	YES	3	110	315	48	930	48
	RPI001_685651	YES	4	696	310	94	925	14
	RPI001_25361	YES	5	411	819	83	234	94
Dataset 2	RP5-1085F17	YES	1	396	116	11	104	116
	RPI001_79181	YES	2	521	276	302	78	15
	RPI001_114047	YES	3	687	567	315	45	63
	RPI001_81047	YES	4	789	330	125	88	17
	RPI001_139850	YES	5	204	360	167	8	65
Dataset 3	RPI001_1036776	YES	1	5	469	3	810	107
	RP11-357C3	YES	2	141	344	16	933	133
	RPI001_878565	YES	3	148	561	50	221	74
	HCG17	YES	4	22	118	4	707	129
	AL139819	YES	5	251	533	34	131	111

#### Finding new LPIs based on known LPIs

**Fig. 2:**
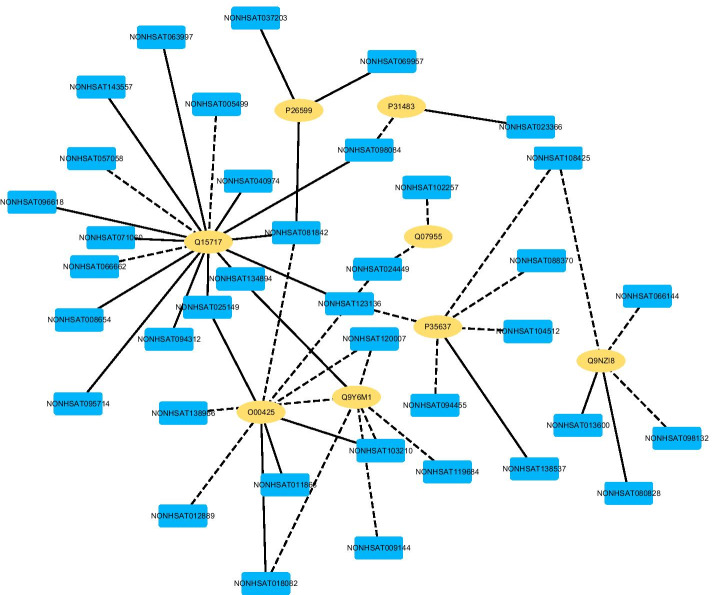
The predicted top 50 LPIs on Dataset 1

**Fig. 3:**
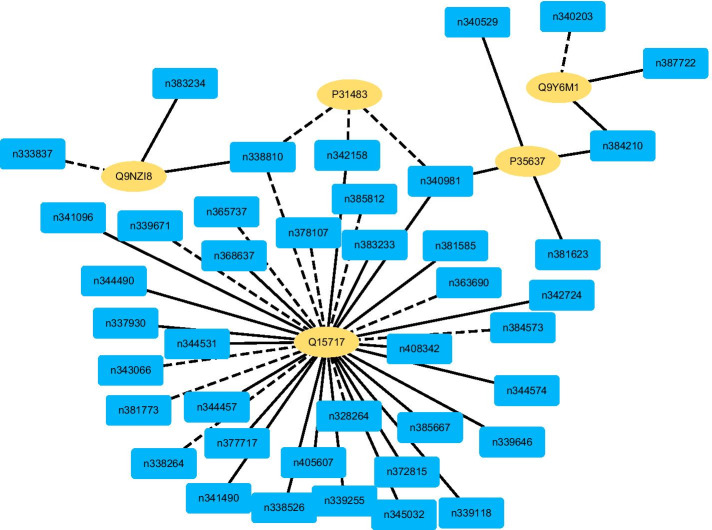
The predicted top 50 LPIs on Dataset 2

**Fig. 4:**
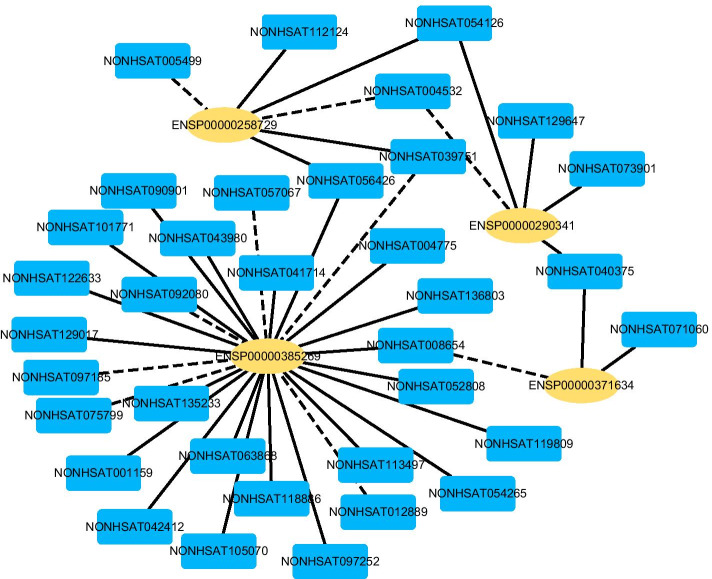
The predicted top 50 LPIs on Dataset 3

**Fig. 5:**
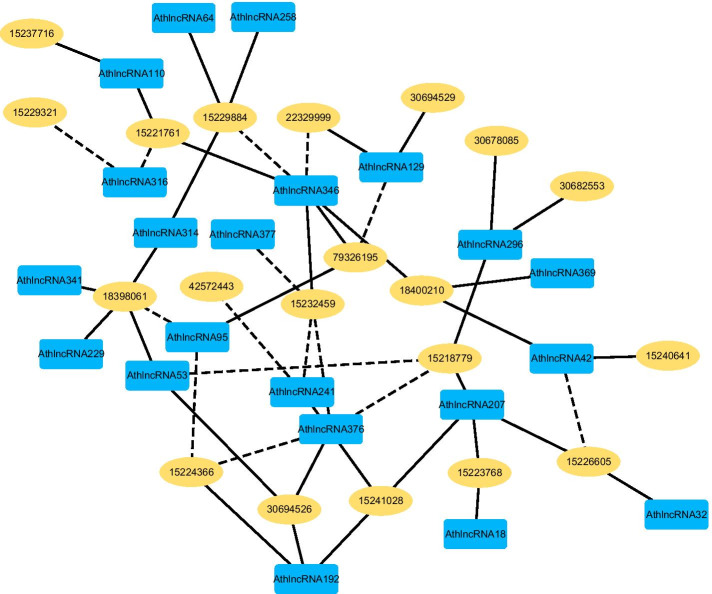
The predicted top 50 LPIs on Dataset 4

**Fig. 6:**
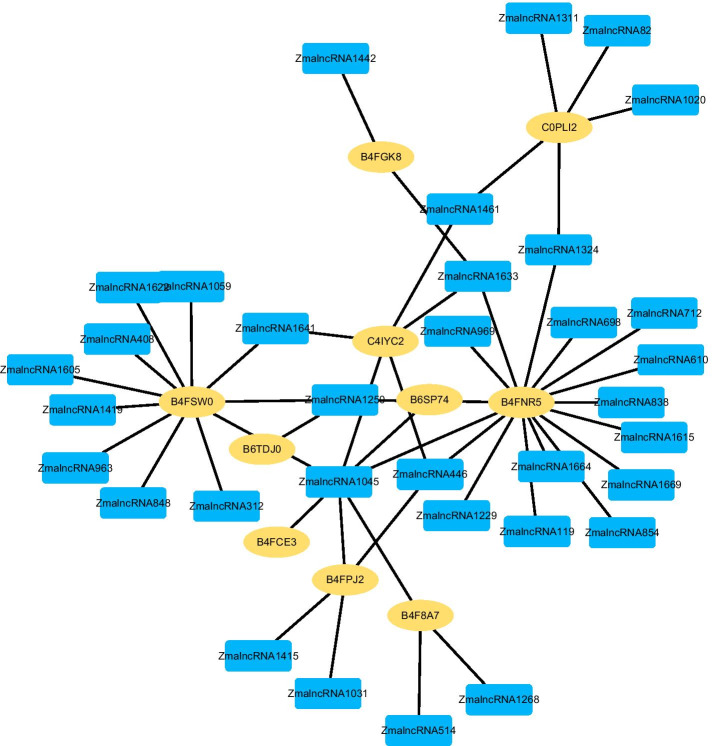
The predicted top 50 LPIs on Dataset 5

We further infer new LPIs based on LPI-deepGBDT. We rank all lncRNA–protein pairs based on the computed average interaction probabilities. Figures 2, 3, 4, 5 and 6 give the predicted 50 LPIs with the highest interaction scores. In the five figures, black dotted lines and solid lines represent unknown and known LPIs obtained from LPI-deepGBDT, respectively. Gold ovals denote proteins, deep sky blue rounded rectangles denote RNA.

There are 55,165, 74,340, 26,730, 3,815, and 71,568 known and unknown lncRNA–protein pairs on given five datasets, respectively. We observe that unknown lncRNA–protein pairs between NONHSAT023366 (RAB30-AS1) and O00425, n378107 (NONHSAT007673, GAS5) and Q15717, NONHSAT143568 (LINC-01572) and P35637, AthlncRNA376 (TCONS_00057930) and O22823, and ZmalncRNA530 (TCONS_00007931) and C0PLI2, which are predicted to have the highest association scores on the five datasets, are ranked as 1, 3, 1, 6, and 113, respectively.

lncRNA GAS5 has close linkages with multiple complex diseases. The lncRNA is a repressor of the glucocorticoid receptors associated with growth arrest and starvation [46]. It is downregulated in breast cancer [47]. It cam also promote microglial inflammatory response in Parkinson’s disease [48], control apoptosis in non-small-cell lung cancer [49] and prostate cancer cell [50]. Its decreased expression indicates a poor prognosis in cervical cancer [51] and gastric cancer [52].

Q15717 increases the stability of mRNA and mediates the CDKN2A anti-proliferative activity and regulates p53/TP53 expression. It increases the leptin mRNA’s stability and is involved in embryonic stem cells differentiation. In dataset 2, GAS5 have been validated to interact with P35637, and Q13148. P35637 plays an important role in diverse cellular processes including transcription regulation, DNA repair and damage response, RNA transport, and RNA splicing. It helps RNA transport, mRNA stability and synaptic homeostasis in neuronal cells. Q13148 plays a crucial role in maintaining mitochondrial homeostasis. It participates in the formation and regeneration of normal skeletal muscle, negatively regulates the expression of CDK6. The three proteins are RNA-binding proteins and have in part similar biological functions. Therefore, we infer that Q15717 may be the corresponding protein of GAS5.

## Discussion and further research

lncRNAs regulate many important biological processes. They have close relationships with multiple human complex diseases. However, most of them are not annotated because of the poor evolutionary conservation. Recent researches suggest that lncRNAs implement their functions by binding to the corresponding proteins. Therefore, it is a significant work to infer potential interactions between lncRNAs and proteins. Various computational methods were designed to identify new LPIs. These models improved LPI prediction and found many potential linkages between the two entities. The predicted LPIs with higher rankings are worthy of further biomedical experimental validation.

In this manuscript, we explore an LPI identification framework (LPI-deepGBDT) based on a feed-forward deep architecture with GBDTs. First, three LPI datasets and two plant datasets are retrieved. Second, the biological features of lncRNAs and proteins are selected via Pyfeat and BioProt, respectively. Third, the features are reduced based on dimensional reduction technique and concatenated to depict an lncRNA–protein pair. Finally, a multi-layered deep framework is developed to find the potential relationships between the two entities. We compare LPI-deepGBDT with five classical LPI discovery methods, LPI-BLS, LPI-CatBoost, PLIPCOM, LPI-SKF and LPI-HNM, on the five datasets under three cross validations. The results demonstrate the superior classification ability of LPI-deepGBDT. Case studies are further implemented to conduct interaction prediction for new lncRNAs (or proteins) or based on known LPIs.

LPI-deepGBDT computes the best performance on the collected five LPI datasets. It may be in large part due to the following features. First, LPI-deepGBDT fuses multiple biological features. Second, the constructed multi-layered deep framework with non-differentiable components helps to distributedly represent the outputs in intermediate layers. Thirdly, the update procedure for each intermediate layer can reduce the global loss by updating its pseudo-label and reducing the loss in the previous layer. Finally, the random noises added in the loss function can better map the neighbor training samples to right manifold.

In the future, we will collect multiple LPI datasets from different species to better mine the relevances between lncRNAs and proteins for different species. More importantly, we will develop more effective ensemble learning model to improve the performance of LPI prediction.

## Data Availability

Source codes and datasets are freely available for download at https://github.com/plhhnu/LPI-deepGBDT.

## Abbreviations

LPI-deepGBDT: Feed-forward deep architecture based on gradient boosting decision trees used to discover unobserved LPIs
LPI: Long noncoding RNA–protein interaction
GBDT: Gradient boosting decision trees
lncRNAs: Long noncoding RNAs
CVs: Cross validations

## References

[1] Deng L, Wang J, Xiao Y, Wang Z, Liu H (2018). Accurate prediction of protein-lncrna interactions by diffusion and hetesim features across heterogeneous network. BMC Bioinform..

[2] Liu Z-P (2020). Predicting lncrna-protein interactions by machine learning methods: a review. Curr. Bioinform..

[3] Chen X, Sun Y-Z, Guan N-N, Qu J, Huang Z-A, Zhu Z-X, Li J-Q (2019). Computational models for lncrna function prediction and functional similarity calculation. Brief. Funct. Genom..

[4] Chen X, Yan CC, Zhang X, You Z-H (2017). Long non-coding rnas and complex diseases: from experimental results to computational models. Brief. Bioinform..

[5] Wang, W., Dai, Q., Li, F., Xiong, Y., Wei, D.-Q.: Mlcdforest: multi-label classification with deep forest in disease prediction for long non-coding rnas. Brief. Bioinform. (2020) 10.1093/bib/bbaa104 32520339

[6] Zhang X, Zhou Y, Mehta KR, Danila DC, Scolavino S, Johnson SR, Klibanski A (2003). A pituitary-derived meg3 isoform functions as a growth suppressor in tumor cells. J. Clin. Endocrinol. Metabol..

[7] Pibouin L, Villaudy J, Ferbus D, Muleris M, Prospéri M-T, Remvikos Y, Goubin G (2002). Cloning of the mrna of overexpression in colon carcinoma-1: a sequence overexpressed in a subset of colon carcinomas. Cancer Genet. Cytogenet..

[8] Cui, Z., Ren, S., Lu, J., Wang, F., Xu, W., Sun, Y., Wei, M., Chen, J., Gao, X., Xu, C., et al.: The prostate cancer-up-regulated long noncoding rna plncrna-1 modulates apoptosis and proliferation through reciprocal regulation of androgen receptor. In: Urologic Oncology: Seminars and Original Investigations, vol. 31, pp. 1117–1123. Elsevier (2013) 10.1016/j.urolonc.2011.11.030 22264502

[9] Chen X, Yan G-Y (2013). Novel human lncrna-disease association inference based on lncrna expression profiles. Bioinformatics.

[10] van Poppel H, Haese A, Graefen M, de la Taille A, Irani J, de Reijke T, Remzi M, Marberger M (2012). The relationship between prostate cancer gene 3 (pca3) and prostate cancer significance. BJU Int..

[11] Yang Z, Zhou L, Wu L-M, Lai M-C, Xie H-Y, Zhang F, Zheng S-S (2011). Overexpression of long non-coding rna hotair predicts tumor recurrence in hepatocellular carcinoma patients following liver transplantation. Ann. Surg. Oncol..

[12] Wang, W., Guan, X., Khan, M.T., Xiong, Y., Wei, D.-Q.: Lmi-dforest: a deep forest model towards the prediction of lncrna-mirna interactions. Comput. Biol. Chem. 107406 (2020) 10.1016/j.compbiolchem.2020.107406 33120126

[13] Li Y, Sun H, Feng S, Zhang Q, Han S, Du W (2021). Capsule-lpi: a lncrna-protein interaction predicting tool based on a capsule network. BMC Bioinform..

[14] Li, A., Ge, M., Zhang, Y., Peng, C., Wang, M.: Predicting long noncoding rna and protein interactions using heterogeneous network model. Biomed. Res. Int. 2015 (2015) 10.1155/2015/671950 PMC4709602 26839884

[15] Zhou Y-K, Shen Z-A, Yu H, Luo T, Gao Y, Du P-F (2020). Predicting lncrna-protein interactions with mirnas as mediators in a heterogeneous network model. Front Genet..

[16] Yang J, Li A, Ge M, Wang M (2016). Relevance search for predicting lncrna-protein interactions based on heterogeneous network. Neurocomputing.

[17] Zhao Q, Yu H, Ming Z, Hu H, Ren G, Liu H (2018). The bipartite network projection-recommended algorithm for predicting long non-coding rna-protein interactions. Mol. Therapy-Nucleic Acids.

[18] Ge M, Li A, Wang M (2016). A bipartite network-based method for prediction of long non-coding rna-protein interactions. Genom. Proteom. Bioinform..

[19] Xie G, Wu C, Sun Y, Fan Z, Liu J (2019). Lpi-ibnra: long non-coding rna-protein interaction prediction based on improved bipartite network recommender algorithm. Front. Genet..

[20] Zhang W, Qu Q, Zhang Y, Wang W (2018). The linear neighborhood propagation method for predicting long non-coding rna-protein interactions. Neurocomputing.

[21] Zhou Y-K, Hu J, Shen Z-A, Zhang W-Y, Du P-F (2020). Lpi-skf: predicting lncrna-protein interactions using similarity kernel fusions. Front. Genet..

[22] Chen Y, Fu X, Li Z, Peng L, Zhuo L (2021). Prediction of lncrna-protein interactions via the multiple information integration. Front. Bioeng. Biotechnol..

[23] Peng L, Liu F, Yang J, Liu X, Meng Y, Deng X, Peng C, Tian G, Zhou L (2020). Probing lncrna-protein interactions: data repositories, models, and algorithms. Front. Genet..

[24] Liu H, Ren G, Hu H, Zhang L, Ai H, Zhang W, Zhao Q (2017). Lpi-nrlmf: lncrna-protein interaction prediction by neighborhood regularized logistic matrix factorization. Oncotarget.

[25] Zhao Q, Zhang Y, Hu H, Ren G, Zhang W, Liu H (2018). Irwnrlpi: integrating random walk and neighborhood regularized logistic matrix factorization for lncrna-protein interaction prediction. Front. Genet..

[26] Zhang T, Wang M, Xi J, Li A (2018). Lpgnmf: predicting long non-coding rna and protein interaction using graph regularized nonnegative matrix factorization. IEEE/ACM Trans. Comput. Biol. Bioinf..

[27] Zhang W, Yue X, Tang G, Wu W, Huang F, Zhang X (2018). Sfpel-lpi: sequence-based feature projection ensemble learning for predicting lncrna-protein interactions. PLoS Comput. Biol..

[28] Fan X-N, Zhang S-W (2019). Lpi-bls: predicting lncrna-protein interactions with a broad learning system-based stacked ensemble classifier. Neurocomputing.

[29] Deng L, Yang W, Liu H (2019). Predprba: prediction of protein-rna binding affinity using gradient boosted regression trees. Front. Genet..

[30] Wekesa JS, Meng J, Luan Y (2020). Multi-feature fusion for deep learning to predict plant lncrna-protein interaction. Genomics.

[31] Shen, Z.-A., Luo, T., Zhou, Y.-K., Yu, H., Du, P.-F.: Npi-gnn: predicting ncrna-protein interactions with deep graph neural networks. Brief. Bioinform. (2021) 10.1093/bib/bbab051 33822882

[32] Feng, J., Yang, Y., Zhou, Z.H.: Multi-layered gradient boosting decision trees (2018)

[33] Xie C, Yuan J, Li H, Li M, Zhao G, Bu D, Zhu W, Wu W, Chen R, Zhao Y (2014). Noncodev4: exploring the world of long non-coding rna genes. Nucleic Acids Res..

[34] Yuan J, Wu W, Xie C, Zhao G, Zhao Y, Chen R (2014). Npinter v2. 0: an updated database of ncrna interactions. Nucleic Acids Res..

[35] Consortium U (2019). Uniprot: a worldwide hub of protein knowledge. Nucleic Acids Res..

[36] Zheng X, Wang Y, Tian K, Zhou J, Guan J, Luo L, Zhou S (2017). Fusing multiple protein–protein similarity networks to effectively predict lncrna-protein interactions. BMC Bioinform..

[37] Bai Y, Dai X, Ye T, Zhang P, Yan X, Gong X, Liang S, Chen M (2019). Plncrnadb: a repository of plant lncrnas and lncrna-rbp protein interactions. Curr. Bioinform..

[38] Muhammod R, Ahmed S, Md Farid D, Shatabda S, Sharma A, Dehzangi A (2019). Pyfeat: a python-based effective feature generation tool for dna, rna and protein sequences. Bioinformatics.

[39] Márquez, B., Amaya, J.C.: Bioprot contenedor autónomo de residuos biológicos. Revista colombiana de tecnologias de avanzada **1**(33) (2019)

[40] Ding C, Wang D, Ma X, Li H (2016). Predicting short-term subway ridership and prioritizing its influential factors using gradient boosting decision trees. Sustainability.

[41] Shi Z, Chu Y, Zhang Y, Wang Y, Wei D-Q (2020). Prediction of blood–brain barrier permeability of compounds by fusing resampling strategies and extreme gradient boosting. IEEE Access.

[42] Friedman, J.H.: Greedy function approximation: a gradient boosting machine. Ann. Stat. 1189–1232 (2001)

[43] Jiao Y, Du P (2016). Performance measures in evaluating machine learning based bioinformatics predictors for classifications. Quant. Biol..

[44] Nabet BY, Qiu Y, Shabason JE, Wu TJ, Yoon T, Kim BC, Benci JL, DeMichele AM, Tchou J, Marcotrigiano J (2017). Exosome rna unshielding couples stromal activation to pattern recognition receptor signaling in cancer. Cell.

[45] Tan C, Cao J, Chen L, Xi X, Wang S, Zhu Y, Yang L, Ma L, Wang D, Yin J (2019). Noncoding rnas serve as diagnosis and prognosis biomarkers for hepatocellular carcinoma. Clin. Chem..

[46] Kino T, Hurt DE, Ichijo T, Nader N, Chrousos GP (2010). Noncoding rna gas5 is a growth arrest-and starvation-associated repressor of the glucocorticoid receptor. Sci. Signal..

[47] Mourtada-Maarabouni M, Pickard M, Hedge V, Farzaneh F, Williams G (2009). Gas5, a non-protein-coding rna, controls apoptosis and is downregulated in breast cancer. Oncogene.

[48] Xu W, Zhang L, Geng Y, Liu Y, Zhang N (2020). Long noncoding rna gas5 promotes microglial inflammatory response in parkinsons disease by regulating nlrp3 pathway through sponging mir-223-3p. Int. Immunopharmacol..

[49] Shi X, Sun M, Liu H, Yao Y, Kong R, Chen F, Song Y (2015). A critical role for the long non-coding rna gas5 in proliferation and apoptosis in non-small-cell lung cancer. Mol. Carcinog..

[50] Pickard M, Mourtada-Maarabouni M, Williams G (2013). Long non-coding rna gas5 regulates apoptosis in prostate cancer cell lines. Biochimica et Biophysica Acta.

[51] Cao S, Liu W, Li F, Zhao W, Qin C (2014). Decreased expression of lncrna gas5 predicts a poor prognosis in cervical cancer. Int. J. Clin. Exp. Pathol..

[52] Sun M, Jin F-Y, Xia R, Kong R, Li J-H, Xu T-P, Liu Y-W, Zhang E-B, Liu X-H, De W (2014). Decreased expression of long noncoding rna gas5 indicates a poor prognosis and promotes cell proliferation in gastric cancer. BMC Cancer.

